# Chromosome painting in three-toed sloths: a cytogenetic signature and ancestral karyotype for Xenarthra

**DOI:** 10.1186/1471-2148-12-36

**Published:** 2012-03-19

**Authors:** Nathália F Azevedo, Marta Svartman, Andrea Manchester, Nádia de Moraes-Barros, Roscoe Stanyon, Angela M Vianna-Morgante

**Affiliations:** 1 Departamento de Genética e Biologia Evolutiva, Instituto de Biociências, Universidade de São Paulo, Rua do Matão 277, Cidade Universitária, São Paulo, SP 05408-090, Brazil; 2 Departamento de Biologia Geral, Instituto de Ciências Biológicas, Universidade Federal de Minas Gerais, Avenida Antônio Carlos 6627, Belo Horizonte, MG 31270-901, Brazil; 3 Department of Evolutionary Biology, University of Florence, Via del Proconsolo 12, Florence 50122, Italy

## Abstract

**Background:**

Xenarthra (sloths, armadillos and anteaters) represent one of four currently recognized Eutherian mammal supraorders. Some phylogenomic studies point to the possibility of Xenarthra being at the base of the Eutherian tree, together or not with the supraorder Afrotheria. We performed painting with human autosomes and X-chromosome specific probes on metaphases of two three-toed sloths: *Bradypus torquatus *and *B. variegatus*. These species represent the fourth of the five extant Xenarthra families to be studied with this approach.

**Results:**

Eleven human chromosomes were conserved as one block in both *B. torquatus *and *B. variegatus*: (HSA 5, 6, 9, 11, 13, 14, 15, 17, 18, 20, 21 and the X chromosome). *B. torquatus*, three additional human chromosomes were conserved intact (HSA 1, 3 and 4). The remaining human chromosomes were represented by two or three segments on each sloth. Seven associations between human chromosomes were detected in the karyotypes of both *B. torquatus *and *B. variegatus*: HSA 3/21, 4/8, 7/10, 7/16, 12/22, 14/15 and 17/19. The ancestral Eutherian association 16/19 was not detected in the *Bradypus *species.

**Conclusions:**

Our results together with previous reports enabled us to propose a hypothetical ancestral Xenarthran karyotype with 48 chromosomes that would differ from the proposed ancestral Eutherian karyotype by the presence of the association HSA 7/10 and by the split of HSA 8 into three blocks, instead of the two found in the Eutherian ancestor. These same chromosome features point to the monophyly of Xenarthra, making this the second supraorder of placental mammals to have a chromosome signature supporting its monophyly.

## Background

**Figure 1:**
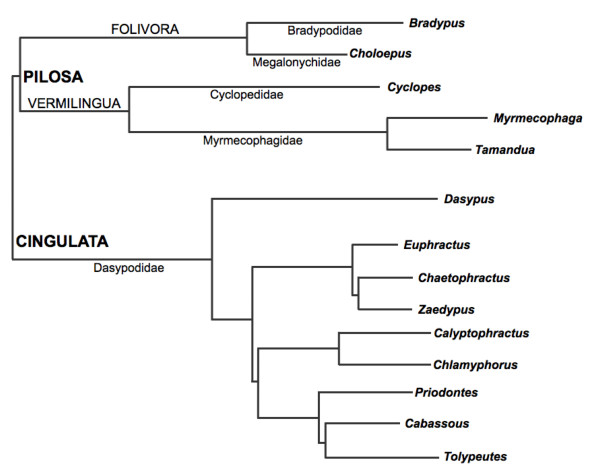
**Phylogenetic relationships of extant xenarthran genera based on current molecular data**. Topology adapted from [63].

Xenarthra, which reunites sloths, armadillos and anteaters, is one of the four currently recognized Eutherian mammal supraorders, together with Afrotheria, Euarchontoglires and Laurasiatheria. The group has as its main morphological synapomorphy used to name it the presence of xenarthrous vertebrae, additional articulations between the dorsal and lumbar vertebrae [1]. Morphological and molecular data support the monophyly of Xenarthra [2-7], which is composed of two living orders: Cingulata, formed by the family Dasypodidae, with 21 species of armadillos; and Pilosa, composed by the suborders Vermilingua, with two families - Cyclopedidae and Myrmecophagidae, and four species of anteaters, and Folivora, with two families - Bradypodidae and Megalonychidae, and six species of sloths [8] (Figure 1).

Xenarthra has lately been the focus of interest because of its possible position at the base of the Eutherian phylogenetic tree. Phylogenies based on morphological data and some molecular studies put Xenarthra at the base of the tree [9]. A second hypothesis, supported by molecular data, puts Afrotheria in the most basal position [6,10-14]. A third possibility, mainly based on molecular analyses and phylogenomics, considers Afrotheria and Xenarthra as sister-groups, forming the clade Atlantogenata, which would be on the base of the Eutherian tree [3,15-21].

### Extant species of sloths

The living species of sloths are grouped into two families [8]. Megalonychidae or the two-toed sloths are represented by a single genus, *Choloepus*, and two species, *C. hoffmanni *and *C. didactylus*. This family is restricted to the Neotropical forests of Central America and northern South America [22]. Bradypodidae or three-toed sloths reunites the four species of the genus *Bradypus. B. variegatus *is found in the Amazon and in the Brazilian Atlantic forest, Caatinga and Cerrado biomes. *B. torquatus *is endemic to the Brazilian Atlantic forest, *B. tridactylus *lives in the Amazon [22] and the recently described *B. pygmaeus *is endemic to the island of Escudo de Veraguas, on the Caribbean coast of Panama [23]. According to the red list of the International Union for Conservation of Nature [24], two sloth species are included in a threatened category; *B. pygmaeus *is listed as critically endangered with decreasing population size, and *B. torquatus *is a vulnerable species due to the degradation and fragmentation of the area where it occurs, the Atlantic forest.

While the monophyly of Folivora (sloths) is highly supported, detailed analyses within the sloth clade indicated the diphyletic origin of *Choloepus *and *Bradypus *[3,25-27].

### Cytogenetics of Xenarthra

The chromosomes of Xenarthra are still poorly studied and most of the described karyotypes were analyzed after conventional staining, without banding patterns [28-30]. The diploid numbers in Xenarthra range from 2n = 38 in the armadillo *Tolypeutes matacus *to 2n = 65 in the sloth *Choloepus didactylus *[29,31]. The diploid numbers range from 2n = 54 to 2n = 64 in the anteaters and from 2n = 38 to 2n = 64 in the armadillos [29,30,32].

In sloths, karyotypic studies showed a complex picture. The karyotypes described for the genus *Choloepus *have a diploid number ranging from 2n = 49 to 2n = 65, which may actually belong to cryptic species not identified as yet [31,33-36] and Y/autosome translocations have been reported in *Choloepus hoffmanni *with 2n = 49 [37] and in *C. didactylus *with 2n = 65 [31]. The diploid numbers of *Bradypus *species are better characterized: *Bradypus torquatus *showed 2n = 50 [38,39], *Bradypus variegatus *had 2n = 54 [34,38,40] and *Bradypus tridactylus *had 2n = 52 [31,33]. The karyotype of *B. pygmaeus *has not been described.

### Molecular cytogenetics and chromosome painting

Chromosome painting is a very useful tool in phylogenetic studies because chromosome rearrangements are considered rare genomic changes with low levels of homoplasy [41]. The resolution of the technique is sufficient to allow the reconstruction of lineages that show relationships among species, families and orders, often revealing characteristic chromosome signatures of each lineage [42]. Based on interspecific chromosomal painting using human chromosomes as probes, there are currently two main proposals for a hypothetical ancestral Eutherian karyotype (AEK). An AEK with 2n = 48 would be composed of human chromosomes HSA 1, 2p, 2q, 3/21, 4/8p, 5, 6, 7a, 7b/16p, 8q, 9, 10p, 10q, 11, 12/22 twice, 13, 14/15, 16q/19q, 17, 18, 19p, 20 and X [43,44]. Alternatively, an AEK with 2n = 46, differing only by an additional association HSA 10p/12/22, has also been suggested (reviewed in [42]).

Painting with human chromosome has been performed in three of the five recognized families of Xenarthra: in the lesser anteater (*Tamandua tetradactyla*), in two species of two-toed sloths (*Choloepus didactylus *and *C. hoffmanni*), and in two species of armadillos (*Dasypus novemcinctu*s and *Euphractus sexcinctus*) [36,45,46]. In this work, we performed painting with human chromosome-specific probes in two species of three-toed sloths: *Bradypus torquatus *and *B. variegatus*. These species represent the fourth family of Xenarthra analyzed with this approach, which enabled us to hypothesize on an ancestral Xenarthran karyotype and to compare it to the putative ancestral Eutherian complement. Our data also allowed us to discuss chromosome signatures supporting the subordinal levels of classification in Xenarthra and Folivora phylogeny.

## Results

### B. torquatus

**Figure 2:**
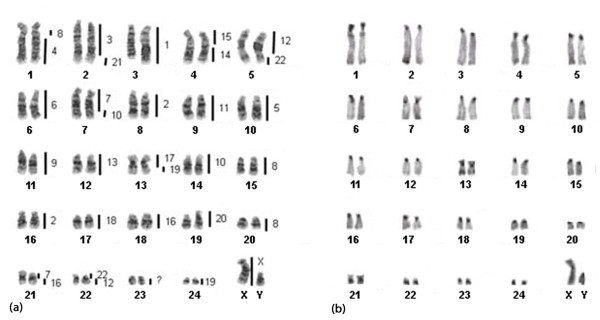
**Correspondence between human chromosomes and (a) the G-banded karyotype of a male *Bradypus torquatus *(2n = 50); (b) C-banded karyotype of a male *Bradypus torquatus***. Each sloth chromosome segment was painted by the human chromosome indicated to the right.

**Figure 3:**
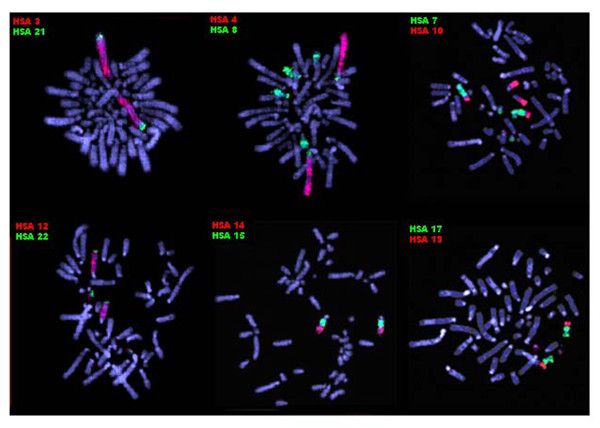
**Partial metaphases of *Bradypus torquatus *(2n = 50) after FISH with human chromosome-specific paints**. The probes used are indicated for each experiment. The green signals were produced by biotin-labeled probes detected with FITC-conjugated avidin and the red signals, by digoxigenin-labeled probes detected with rhodamine-conjugated antidigoxigenin. The chromosomes were counterstained with DAPI.

Interspecies hybridization results were obtained for all the human probes except the Y in *B. torquatus *chromosomes (BTO). A total of 32 conserved segments between human (HSA) and BTO were observed (Figure 2a; Table 1). Fifteen human chromosomes hybridized to a single sloth chromosome or chromosome segment (HSA 1, 3, 4, 5, 6, 9, 11, 13, 14, 15, 17, 18, 20, 21 and the X chromosome). Seven human chromosomes (HSA 2, 7, 10, 12, 16, 19 and 22) labeled two segments each on the sloth karyotype and HSA 8 produced three hybridizations signals in *B. torquatus*. Seven associations between human chromosomes were detected in the karyotype of *B. torquatus*: HSA 3/21, 4/8, 7/10, 7/16, 12/22 (twice), 14/15 and 17/19. Some examples of the hybridization experiments are depicted in Figure 3.

**Table 1:** Correspondence between human and Xenarthran chromosomes based on chromosome painting with human probes

Species	Associations of human autosomes	Human Chromosomes			References
			
		Conserved	Two blocks	Three or more blocks	
*Bradypus torquatus * (2n = 50)	3/21, 4/8, 7/10, 7/16, 12/22(2x), 14/15, 17/19	1, 3, 4, 5, 6, 9, 11, 13, 14, 15, 17, 18, 20, 21, X	2, 7, 10, 12, 16, 19, 22	8	This work

*Bradypus variegates * (2n = 54)	3/21?, 4/8, 7/10, 7/16, 12/22, 12/22/16, 14/15, 17/19	5, 6, 9, 11, 13, 14, 15, 17, 18, 20, 21?, X	1, 2, 3, 4, 7, 10, 12, 19, 22	8, 16	This work

*Bradypus tridactylus * (2n = 52)	2/6, 3/21, 4/8, 7/10, 7/16, 11/19, 12/22, 12/22/16,	1, 5, 9, 11, 13, 14, 15, 17, 18, 20, 21, X	2, 3, 4, 7, 10, 12, 16, 19, 22	6, 8?	inferred from [31,46]

*Choloepus hoffmanni * (2n = 50)	3/21, 4/8, 7/16, 12/22(2x), 14/15, 16/19	1, 3, 4, 5, 6, 9, 10, 11, 13, 14, 15, 17, 18, 20, 21, X	2, 7, 12, 19, 22	8?, 16	[36]

*Choloepus didactylus * (2n = 65)	2/8, 3/21, 4/8, 7/10, 7/16, 12/22 (2x), 14/15	9, 13, 15, 17, 18, 20, 21, X	1, 3, 4, 5, 6, 10, 11, 12, 14, 16, 19, 22	2, 7, 8	[46]

*Dasypus novemcinctus * (2n = 64)	3/21(2x), 4/8, 7/16, 10/12, 12/22(2x), 14/15, 16/19	5, 9, 13, 14, 15, 17, 18, 20, X	1, 4, 6, 7, 10, 11, 16, 19?, 21, 22	2?, 3, 8, 12	[36]

*Euphractus sexcinctus * (2n = 58)	2/8, 3/21, 4/8, 7/10, 7/16, 12/22 (2x), 14/15, 16/19	5, 9, 13, 14, 15, 17, 18, 20, 21, X	1, 6, 7, 10, 11, 16, 19, 22	2, 3, 4, 8, 12,	[45]

*Tamandua tetradactyla * (2n = 54)	1/9, 1/13, 1/19, 2/8, 3/6, 3/21, 4/8, 7/16, 8/17, 12/22(2x), 14/15(2x), 16/19, 3/22, 5/11, 7/20, 20/7/10	9, 10, 13, 17, 18, 20, 21, X	2, 6, 7, 11, 12, 14, 15, 16, 19, 22	1, 3, 4, 5, 8	[36,46]

No hybridization signals were observed on the pericentromeric regions of the sloth chromosomes. Chromosome BTO23, the short arms of BTO1 and BTO4, and a small proximal segment on the long arm of BTO1 were not labeled by any of the human chromosome probes (Figure 2a). Except for BTO 23, these unlabeled regions corresponded to constitutive heterochromatin as revealed by C-banding (Figure 2b).

### B. variegatus

**Figure 4:**
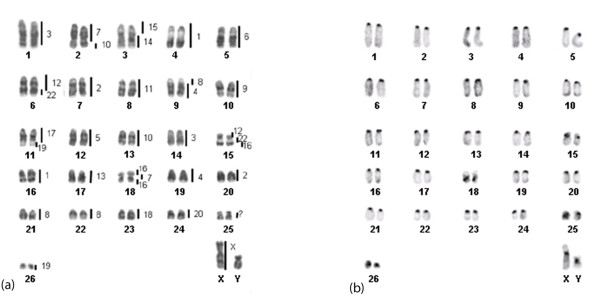
**Correspondence between human chromosomes and (a) the G-banded karyotype of a male *Bradypus variegatus *(2n = 54); (b) C-banded karyotype of a male *B. variegatus***. Each sloth chromosome segment was painted by the human chromosome indicated to the right.

**Figure 5:**
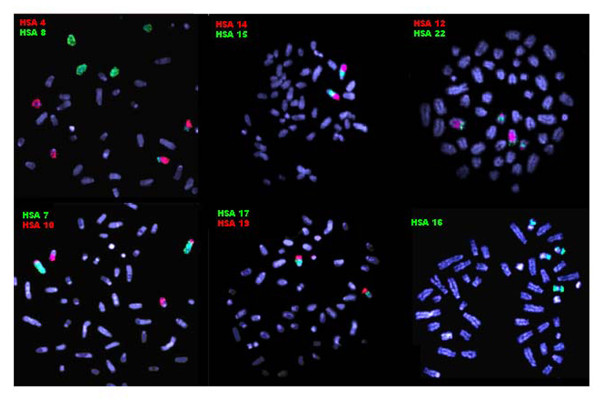
**Partial metaphases of *Bradypus variegatus *(2n = 54) after FISH with human chromosome-specific paints**. The probes used are indicated for each experiment. The green signals were produced by biotin-labeled probes detected with FITC-conjugated avidin and the red signals, by digoxigenin-labeled probes detected with rhodamine-conjugated antidigoxigenin. The chromosomes were counterstained with DAPI.

With the exception of HSA 21, the human autosomal and X chromosome probes hybridized to the *B. variegatus *chromosomes (BVA), yielding a total of 35 conserved segments (Figure 4a; Table 1). The results of some hybridization experiments are shown in Figure 5. Eleven human chromosomes (HSA 5, 6, 9, 11, 13, 14, 15, 17, 18, 20 and the X) hybridized to a single sloth chromosome or chromosome segment and nine human chromosomes (HSA 1, 2, 3, 4, 7, 10, 12, 19 and 22) labeled two segments in different sloth chromosomes Three hybridization signals were obtained on the sloth karyotype after hybridization of HSA 8. HSA 16 also labeled three BVA segments, but two of them on the same chromosome. Seven associations between human chromosomes were observed on the karyotype of *B. variegatus*: HSA 4/8, 7/10, 7/16, 12/22, 12/22/16, 14/15 and 17/19. We could not get hybridization results with the HSA 21 probe and thus the ancestral association HSA 3/21 could not be observed in this species. Nevertheless, it is very likely present on chromosome BVA 1, which was proximally labeled by HSA 3 and had a non-heterochromatic distal segment unlabeled by any human chromosome probe. Besides, after G-banding, BVA1 seems to correspond to BTO 2, which in turn is homologous to HSA 3/21. If these conclusions are accurate, a total of 36, instead of 35, conserved segments are present in the complement of *B. variegatus *after painting with human chromosomes.

No hybridization signals were observed on the pericentromeric regions of the BVA chromosomes, neither were the proximal segments of the short and long arms of BVA 18 or the entire BVA 25 labeled by any human probe (Figure 4a). Excepting for BVA 25, these segments were heterochromatic as revealed by C-banding (Figure 4b).

## Discussion

Our data on chromosome painting with human probes in two three-toed sloth species, *Bradypus torquatus *(2n = 50) and *B. variegatus *(2n = 54) raises to four the number of Xenarthra families studied with this approach, leaving only the chromosomes of the monospecific family Cyclopedidae to be analyzed after chromosome painting with human probes.

**Figure 6:**
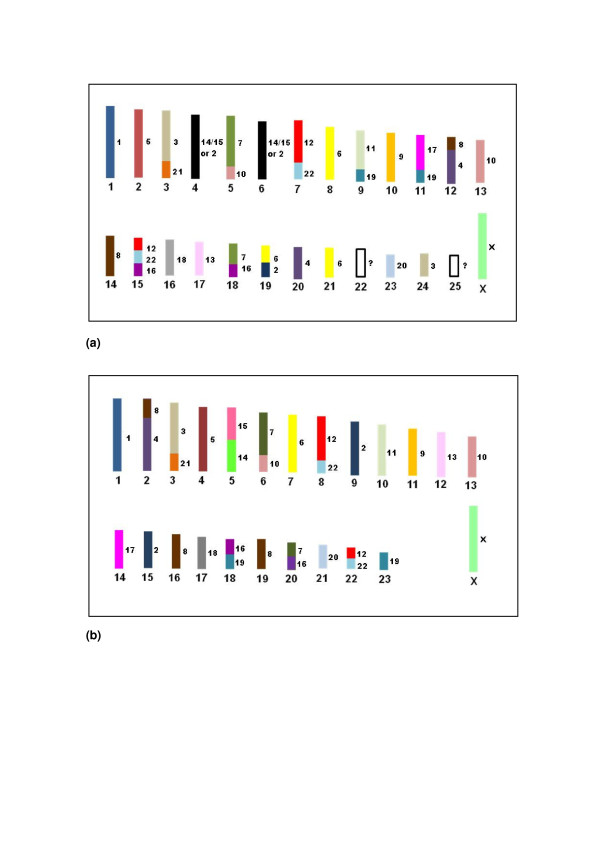
**Schematic representation of the correspondence of human chromosomes to those in (a) *B. tridactylus *karyotype (2n = 52) and (b) the proposed ancestral Xenarthran karyotype (AXK), with 2n = 48**. The human chromosomes are represented by the numbers on the right and by the color code.

The three-toed sloth *Bradypus tridactylus *(2n = 52) had its chromosomes painted with chromosome-specific probes from the two-toed sloth *Choloepus didactylus *(CDI) [31] and human chromosome probes were hybridized to the chromosomes of CDI [46]. Except for CDI 18, 20 and 30, which correspond, respectively, to HSA 2/8, 14 and 11, all CDI probes labeled chromosome segments in *B. tridactylus*. These results allowed us to infer the correspondence between the human chromosomes and those of *B. tridactylus *(Figure 6a; Table 1). In *B. tridactylus*, at least 35 segments would result from chromosome painting with human probes (Figure 6a).

Painting with human chromosome-specific probes revealed features common to the karyotypes of the two sloth species studied herein and also in the inferred human painted karyotype of *B. tridactylus *(Table 1; Figure 6a): (a) the associations HSA 3/21, 4/8, 7/10, 7/16, 12/22, 14/15 and 17/19, (b) the conservation of HSA 5, 9, 11, 13, 14, 15, 17, 18, 20 and X, (c) two pairs labeled by HSA 2, 7, 10, 12, 19 and 22. In addition, the ancestral association HSA 16/19, present in most Eutherians, was absent in the three-toed sloths. HSA 8 was divided into three segments in *B. torquatus *and *B. variegatus*, as already shown in all the other Xenarthra species analyzed so far [36,45,46], with the exception of *B. tridactylus*. Nevertheless, the CDI 18 probe, which corresponds to HSA 2/8, did not hybridize in *B. tridactylus *[31] and it is thus likely that HSA 8 is also divided into three blocks in this species.

The HSA 17/19 association present in the three *Bradypus *species has not been found in any other species of Eutheria. We thus propose that this association represents a chromosome signature of the genus *Bradypus*, supporting the monophyly of the group, already indicated by molecular data [47].

The conservation of HSA 1 as only one chromosome and the split of HSA 16 in two blocks are shared by *B. tridactylus *and *B. torquatus *and are present in the proposed Eutherian ancestral karyotypes, representing thus symplesiomorphies. On the other hand, the presence of HSA 3 and 4 in two blocks and of the association HSA 12/22/16, common to *B. variegatus *and *B. tridactylus*, are derived characteristics, absent in the ancestral karyotype. The fission of HSA 3 and 4 was already reported in other species of Eutheria, including Xenarthra [36,46,48,49], but the HSA 12/22/16 association was not observed in any other Xenarthra species or outgroup. We thus suggest that this association represents a chromosome synapomorphy common to *B. variegatus *and *B. tridactylus*. Reciprocal chromosome painting would allow the precise identification of the chromosome segments involved in the HSA 12/22/16 associations and the fissions of HSA 3 and 4 in both species. Our results coupled with previously reported data [31,46] indicate that *B. variegatus *and *B. tridactylus *have more similar karyotypes than each of them has to *B. torquatus*, sharing even the derived HSA 12/22/16 association. These results corroborate the recent published *Bradypus *molecular phylogeny, which indicated *B. torquatus *as a basal lineage within the genus [47]. The divergence between *B. torquatus *and the remaining *Bradypus *species occurred around 12 mya, *B. tridactylus *and *B. variegatus *being sister species, which diverged 6 mya. This finding gives some support to the notion that *B. torquatus *may be a different genus, as already suggested on the basis of morphological and molecular analyses [50,51].

### Suborder Folivora (sloths)

In sloths, the ancestral association HSA 16/19 was previously detected in *C. hoffmanni *[36]. This association was absent in *C. didactylus *[46] and we did not observe it in *B. torquatus, B. variegatus *or *B. tridactylus*. The absence of this ancestral Eutherian association in four sloth species is noteworthy because it is present in the great majority of placental mammals. This association may have been lost in the common ancestor of the genus *Bradypus *and in the lineage of *C. didactylus *or it may have disappeared in the common ancestor of the sloths and latter reappeared in the *C. hoffmanni *lineage. HSA 16q/19q is the only Eutherian ancestral association derived from centric fusion [46]. Breakpoints in centromeric regions tend to be recurrent [52], which could explain the loss of the association twice, in *Bradypus *and in *C. didactylus*, or even its reappearance in *C. hoffmanni*, in the case that it was lost in a common ancestor of all the sloths.

No chromosome synapomorphy uniting the two genera of sloths was detected. This may indicate that no significant chromosome rearrangements accompanied the divergence of the suborder Folivora or that they do not share a recent common ancestor. Phylogenetic analysis based on morphological data of both extinct and extant species confirmed the grouping of *Choloepus *sloths to the Megalonychidae and placed *Bradypus *as the sister-taxon of all remaining sloths [26,27]. Molecular phylogenetic studies also indicated that each extant sloth species shares a more recent common ancestor with an extinct ground sloth species than with each other [50,53-55]. Other molecular phylogenetic studies including only extant sloth species indicated a very ancient divergence and estimated the separation between the two modern genera at 21 mya, supporting a taxonomic distinction at a high rank [3,25].

*B. torquatus *and *C. hoffmanni*, which have the lowest diploid numbers among the sloths, have the most conserved karyotypes in relation to those proposed as ancestral to Eutheria. They retain intact HSA 1, 3 and 4, each split into two blocks in *B. variegatus *and *C. didactylus*. A strong correlation between the maintenance of the ancestral chromosome corresponding to HSA 1 and low genomic evolution rates, exemplified by dolphins and humans was stressed by [43]. This is indeed the case of *B. torquatus *and *C. hoffmanni *that show the most conserved karyotypes in relation to a putative common ancestral Eutherian karyotype among sloths [36].

### Order Pilosa (anteaters and sloths)

The division of HSA 12 in two blocks seems common to the order Pilosa (anteaters and sloths), while this chromosome is represented by three blocks in two species of armadillos [36,45]. Two pairs corresponding to HSA 12 are present in the karyotypes of most placental mammals and in the proposed ancestral Eutherian karyotypes [42,43]. The division of this human chromosome in three blocks in the armadillos is thus derived, whereas its presence in two segments in Pilosa is a symplesiomorphy. The monophyly of the order Pilosa is strongly supported by molecular data [2,3,5], but chromosomal synapomorphies were not detected in the group, suggesting that the divergence of the clade was not accompanied by important chromosome rearrangements.

### Ancestral Xenarthran karyotype

Besides the three *Bradypus *species discussed herein, five other species of Xenarthra were analyzed after painting with human chromosomes: *Tamandua tetradactyla *(lesser anteater), *Dasypus novemcinctus *(nine-banded armadillo), *Euphractus sexcinctus *(six-banded armadillo), *Choloepus hoffmanni *(Hoffmann's two-toed sloth), and *C. didactylus *(two-toed sloths) [36,45,46]. Our results together with those previously reported for other Xenarthra show some shared features among all the studied species: (a) the conservation of HSA 9, 13, 17, 18, 20 and X, (b) two pairs sharing homology with HSA 19 and 22 and (c) the presence of the ancestral Eutherian associations HSA 3/21, 4/8, 7/16, 12/22 and 14/15 and (d) three chromosome pairs labeled by HSA 8 (Table 1).

Based on the comparisons of the karyotypes of species from the four families of Xenarthra among themselves or with the human karyotype and taking into account the karyotypes of outgroups, we propose an ancestral karyotype for the supraorder Xenarthra (AXK), with 2n = 48 (Figure 6b). This karyotype contains: (a) the associations HSA 3/21, 4/8, 7/10, 7/16, 12/22 (2x), 14/15 and 16/19, (b) the conserved chromosomes HSA 1, 3, 4, 5, 6, 9, 11, 13, 14, 15, 17, 18, 20, 21 and X, (c) two pairs homologous to HSA 2, 7, 10, 12, 16, 19 and 22 and (d) three pairs homologous to HSA 8.

Some chromosomes or chromosome associations of *C. didactylus *(CDI 7, 8, 10, 14, 17, 24, 29, 3/31, 5/13b, 6/22, 9/25 and 26/27) were suggested as ancestral for Xenarthra, based on their presence in at least one Pilosa species and the outgroup, the six-banded armadillo *E. sexcinctus *[31]. These chromosomes correspond to, respectively, HSA 1a, 9, 1b, 17, 13, 20, 7/16, 11/19, 3/21, 7/10, 5, and 16/19. From these ancestral Xenarthran chromosomes, HSA 1 split into two and the HSA 11/19 association are not included in our hypothetical AXK. Instead, HSA 1 would have been conserved as a single chromosome in the ancestral complement, because it was conserved as such in the Eutherian ancestor and in three species of Xenarthra (*C. hoffmanni, B. torquatus*, and *B. tridactylus*), and must have been convergently split in the karyotypes of the other species of the group, as already demonstrated for other placental mammals [43]. We did not include the HSA 11/19 association in the proposed ancestral Xenarthran karyotype, because it is present in just two species of the group (*B. tridactylus *and *E. sexcinctus*), besides being absent from the karyotypes of the outgroups and from the proposed ancestral Eutherian. The remaining chromosomes suggested by [31] are also present in our hypothetical AXK.

The associations HSA 2/8 and 7/10 have been suggested as chromosome signatures of the supraorder Xenarthra [45,46]. These authors detected the association HSA 2/8 in *C. didactylus, T. tetradactyla *and *E. sexcinctus*, but it has not been observed in any other species of the group, including *B. torquatus, B. variegatus *and *B. tridactylus *in the present study. We thus conclude that its presence in *C. didactylus *and *T. tetradactyla*, which present rearranged karyotypes when compared to other Xenarthra, probably results from convergence.

The association HSA 7/10 was reported in *C. didactylus, T. tetradactyla *and *E. sexcinctus *and, by inference, in *B. tridactylus *[45,46]. We also observed this association in the karyotypes of *B. torquatus *and *B. variegatus*. This association is thus present in species representing the four Xenarthra families analyzed to date and was not found in any other Eutherian order, which supports the proposition that it is a chromosome signature of Xenarthra [46]. From all the Xenarthra species analyzed with human chromosome-specific probes, HSA 7/10 was not detected only in *C. hoffmanni *and in *D. novemcinctus *[36]. Nevertheless, these authors noted that a small distal segment of the sloth chromosome painted by HSA 7 was not labeled by any other human probe. It is possible that the unlabeled region corresponds to HSA 10 and was not detected.

The splitting of HSA 8 in three blocks was also proposed as a chromosome synapomorphy of Xenarthra [36]. This characteristic was observed in the karyotypes of the two-toed sloths *C. didactylus *and *C. hoffmanni*, in the anteater *T. tetradactyla *and in the armadillos *D. novemcinctus *and *E. sexcinctus *[36,45,46]. We also observed three HSA 8 labeled chromosome pairs in *B. torquatus *and *B. variegatus*, and this feature is also possibly present in *B. tridactylus*, which lends further support to this being a synapomorphy.

An ancestral Xenarthran karyotype with 2n = 54, similar to the complement of *E. sexcinctus *(2n = 58) was suggested by [45]. The differences in relation to our proposed AXK are: chromosomes HSA 1, 4 and 6, which we considered as conserved, would be divided into two blocks; the association 2/8 would be present, and HSA 2 would be divided into three blocks, instead of two. As discussed above, we favor the presence of a conserved HSA 1 and the absence of the HSA 2/8 association in our proposed AXK. In the currently most accepted versions of the common ancestral eutherian karyotype (AEK), HSA 1, 4 and 6 are conserved and HSA 2 is divided into two [42,43].

HSA 2 painted two chromosomes in the three *Bradypus *species, in *C. hoffmanni *and in *T. tetradactyla*, while three chromosomes were labeled by HSA 2 in *C. didactylus, E. sexcinctus *and possibly in *D. novemcinctus*. We thus believe that the current data favor the idea that HSA 2 was present as two chromosomes in a putative AXK, instead of three, as proposed by [45].

HSA 4 painted two chromosome segments in *B. variegatus, B. tridactylus, D. novemcinctus *and *C. didactylus*, only one chromosome in *C. hoffmanni *and *B. torquatus*, and was split into three in *E. sexcinctus *and *T. tetradactyla *[36,45,46]. *Tamandua tetradactyla *has a relatively rearranged karyotype, as already noted, thus leaving *E. sexcinctus *as the sole species to be considered. This supports our conclusion that HSA 4 was more likely divided into two in the common ancestor of Xenarthra.

HSA 6, considered conserved in the AEK, was intact in *B. torquatus, B. variegatus *and *C. hoffmanni*; it was found in two blocks in *C. didactylus, D. novemcinctus, E. sexcinctus *and *T. tetradactyla *and it was split into three blocks in *B. tridactylus*. With these data, we consider it still uncertain if this chromosome would be represented in the AXK by only one pair, as we propose, or as two blocks, as proposed by [45]. The analysis of additional Xenarthra species promise to shed some light on this issue. For this purpose, data on Dasypodidae will be especially relevant, as this family is still relatively poorly analyzed with chromosome painting: only two out of 21 species had their karyotypes painted.

Our proposed ancestral Xenarthran karyotype differs from that suggested as ancestral to all Eutheria, with 2n = 48 [43,44] by the presence of the association HSA 7/10 and by the split of HSA 8 into three blocks, with only two corresponding blocks in the Eutherian ancestor.

The associations HSA 1/19 and 5/21 were suggested as chromosome signatures of the supraorder Afrotheria [44,56-58]. With the identification of the HSA 7/10 association and the division of HSA 8 in three blocks, Xenarthra becomes the second placental mammal supraorder to have chromosome features pointing to its monophyly. The fusion of the segments corresponding to human chromosomes 7 and 10 and the fission of the segments corresponding to HSA 8 must have occurred in a common Xenarthra ancestor. However, it should be noted that reciprocal chromosome painting, gene mapping or sequencing studies are needed to confirm that the same chromosome segments and breakpoints are involved in the rearrangements of different Xenarthra species.

### The base of the tree

There is still controversy on which clade is on the base of the Eutherian phylogenetic tree, Afrotheria, Xenarthra or a combination of the two [3,9,11,12,15,18,19,59]. The HSA 1/19 association was proposed as a synapomorphy uniting Afrotheria and Xenarthra [42]. This association was found in all the studied species of Afrotheria, but in Xenarthra it was only observed in the lesser anteater *T. tetradactyla *[36,46], the species with the most rearranged karyotype of the group. Moreover, there is no evidence that this association is homologous in the two groups [31,36,46,49]. We did not observe the HSA 1/19 association in the karyotypes of *B. variegatus *and *B. torquatus *and it does not seem to be present in *B. tridactylus*. Thus, there are no good data to support the hypothesis that the 1/19 association is a shared chromosomal characteristic uniting Afrotheria and Xenarthra.

*In silico *comparisons of the genomes of the marsupial *Monodelphis domestica *and the chicken *Gallus gallus *with the human genome (*Ensembl*) reveal an association between HSA 7q and 10p in the opossum chromosome 8 and in the chicken chromosome 1. The HSA 7/10 association seems to be a chromosome feature of Xenarthra, but reciprocal chromosome painting experiments should be performed to verify if homologous chromosome segments are involved in the association in Xenarthra and in the outgroups (marsupial and chicken). The HSA 10 segment involved in the association in Xenarthra is likely to be HSA 10p, as is the case in the marsupial and the chicken. In the karyotypes proposed as ancestral in Eutheria, HSA 10 is split in two ancestral chromosomes (corresponding to HSA 10p and 10q). In Xenarthra the smallest segment corresponding to HSA 10 that probably represents HSA 10p, is associated with HSA 7. If the HSA 7/10 association in Xenarthra and in the outgroups proves to be homologous, it would be a strong indication that this association is ancestral to Eutheria and would support Xenarthra as basal for all placental mammals. The HSA 7/10 association present in the placental mammal ancestor would then have been lost in the lineage leading to the other placental mammals lineages, after the divergence of Xenarthra. If Afrotheria was the first supraorder to diverge from Eutheria or if Xenarthra and Afrotheria were sister-groups, [15,18,19,21], then the HSA 7/10 association would have been lost in the Afrotheria lineage and in the lineage leading to the remaining groups of Eutheria.

After chromosome painting with human probes in species of Xenarthra, the hypothesis that this supraorder is the basal group of Eutheria could not be discarded, as species of the group showed karyotypes very similar to those proposed as ancestral for Eutheria [36]. These data gained further support from the demonstration of two retropositions present in the genomes of all Eutherian clades, to the exclusion of Xenarthra [60].

## Conclusions

Our analysis of chromosome painting in three-toed sloths allowed the proposition of a putative Ancestral Xenarthran Karyotype (AXK) and its comparison with the current versions of the Ancestral Eutherian Karyotype (AEK). We suggest that the association HSA 17/19 is a chromosome signature of the genus *Bradypus*. The association HSA 7/10 and the division of HSA 8 in three blocks would be chromosome features linking all Xenarthra and would be responsible for the differences between the AEK and the AXK. Recent work with chromosome e-painting comparing the genome sequences of *Monodelphis domestica *and *Gallus gallus *with the human genome (*Ensembl*) have already revealed some associations that could be ancestral to all these taxa. Further analyses combining molecular cytogenetics and genome sequencing will ultimately help to define which chromosome features are symplesiomorphic or synapomorphic for different groups.

## Methods

Our sample consisted of animals from Brazil: one male *Bradypus torquatus *(2n = 50) from Santa Luzia, state of Bahia, one female *B. variegatus *(2n = 54) from Itabuna, state of Bahia, and two males of *B. variegatus *(2n = 54), one from Itapecerica da Serra and another from the city of São Paulo, both in the state of São Paulo. The material was collected with a license from the Instituto Brasileiro do Meio Ambiente e dos Recursos Naturais Renováveis (IBAMA, license 032/2005-CGFAU/LIC). Chromosome preparations were obtained from peripheral blood and fibroblasts cultures following conventional methods. G-banding was carried out according to [61] and C-banding was performed according to [62].

Human chromosome-specific probes were obtained by flow sorting, amplified and labeled by degenerate oligonucleotide PCR (DOP-PCR) as already described [44]. The interspecific fluorescent *in situ *hybridization (FISH) experiments were performed with a mixture of 1 μg of the biotin or digoxigenin labeled probes pre-annealed with 1 μg of Human Cot-1 DNA (Invitrogen) and resuspended in 15 μl of hybridization buffer (formamide 50%, 2xSSC, 40 mM phosphate buffer). The probe mixtures were denatured at 98°C for 10 minutes and reannealed at 37°C for 30 minutes before hybridization.

The sloths chromosome preparations were treated for one hour in 2xSSC at 37°C and denatured at 72°C for two minutes. Hybridizations were carried out for seven days at 37°C. Post-hybridization washes and immunodetection were performed as previously described [44] and the slides were mounted with Vectashield Mounting Medium (Vector Laboratories) and 0.8 ng/μl DAPI (Sigma-Aldrich Inc.). Analyses were performed under an epifluorescence Zeiss Axiophot 2 microscope equipped with a CCD camera and the images were processed with ISIS (Metasystems).

## Competing interests

The authors declare that they have no competing interests.

## Authors' contributions

NFA and AMVM conceived and designed the experiments. NFA and AM performed the experiments. NMB collected specimens. RS and AMVM contributed reagents/materials and analysis tools. NFA, MS, AMVM and RS analyzed the data. MS and NFA wrote the paper. AMVM, NMB and RS revised the paper. All the authors read and approved the final manuscript.
